# Demonstrating the undermining of science and health policy after the Fukushima nuclear accident by applying the Toolkit for detecting misused epidemiological methods

**DOI:** 10.1186/s12940-022-00884-6

**Published:** 2022-08-24

**Authors:** Toshihide Tsuda, Yumiko Miyano, Eiji Yamamoto

**Affiliations:** 1 Department of Human Ecology, Graduate School of Environmental and Life Science, Okayama University, 3-1-1 Tsushima-naka, Kita-ku, Okayama, 700-8530 Japan; 2 Department of Epidemiology, Graduate School of Medicine, Dentistry and Pharmaceutical Sciences, Okayama University, Okayama, Japan; 3 Okayama University of Science, Okayama, Japan

**Keywords:** Chernobyl, Thyroid, Cancer, Screening, Overdiagnosis, Ultrasound

## Abstract

It is well known that science can be misused to hinder the resolution (i.e., the elimination and/or control) of a health problem. To recognize distorted and misapplied epidemiological science, a 33-item “Toolkit for detecting misused epidemiological methods” (hereinafter, the Toolkit) was published in 2021. Applying the Toolkit, we critically evaluated a review paper entitled, “Lessons learned from Chernobyl and Fukushima on thyroid cancer screening and recommendations in the case of a future nuclear accident” in *Environment International* in 2021, published by the SHAMISEN (Nuclear Emergency Situations - Improvement of Medical and Health Surveillance) international expert consortium. The article highlighted the claim that overdiagnosis of childhood thyroid cancers greatly increased the number of cases detected in ultrasound thyroid screening following the 2011 Fukushima nuclear accident. However, the reasons cited in the SHAMISEN review paper for overdiagnosis in mass screening lacked important information about the high incidence of thyroid cancers after the accident. The SHAMISEN review paper ignored published studies of screening results in unexposed areas, and included an invalid comparison of screenings among children with screenings among adults. The review omitted the actual state of screening in Fukushima after the nuclear accident, in which only nodules > 5 mm in diameter were examined. The growth rate of thyroid cancers was not slow, as emphasized in the SHAMISEN review paper; evidence shows that cancers detected in second-round screening grew to more than 5 mm in diameter over a 2-year period. The SHAMISEN consortium used an unfounded overdiagnosis hypothesis and misguided evidence to refute that the excess incidence of thyroid cancer was attributable to the nuclear accident, despite the findings of ongoing ultrasound screening for thyroid cancer in Fukushima and around Chernobyl. By our evaluation, the SHAMISEN review paper includes 20 of the 33 items in the Toolkit that demonstrate the misuse of epidemiology. The International Agency for Research on Cancer meeting in 2017 and its publication cited in the SHAMISEN review paper includes 12 of the 33 items in the Toolkit. Finally, we recommend a few enhancements to the Toolkit to increase its utility.

**Supplementary Information:**

The online version contains supplementary material available at 10.1186/s12940-022-00884-6.

## Background

Two major tendencies in the field of environmental epidemiology were pointed out at the beginning of the article by Etzel et al. as hindering the use of research results in the prevention of environmental hazards [1]. First, there is a tendency to emphasize the weaknesses of epidemiological studies with the intention of undermining the work of epidemiology as a method for revealing the human health effects caused by environmental pollution. Another trend is the massive dissemination of misinformation in journals related to environmental epidemiology that is contrary to the scientific evidence, to hinder the resolution of a problem [1].

Distortion and misinformation regarding scientific methods and evidence have been intentionally utilized by industries [2]. Although science can be misused either intentionally, through error, or because of bias [3], a principal method to deflect unwanted policy implications of properly conducted epidemiological studies is to deliberately frame the results in a way that casts doubt and manufactures uncertainty about their validity [1, 2].

An International Network for Epidemiology in Policy (INEP) position statement was focused on conflict-of-interest (COI) and disclosure in epidemiology because such conflicts have been associated with the misuse of epidemiological science [3]. The statement also listed dozens of examples of ways to manage COI that include identification, avoidance, disclosure, and recusal [3]. To recognize distorted and misapplied epidemiological science, techniques used to manipulate epidemiological findings, summarized as part of the INEP Statement in 2020, were expanded and further elucidated in the article entitled, “Toolkit for detecting misused epidemiological methods” (hereinafter, the Toolkit) [4]. This Toolkit consists of 33 items that directly relate to the inappropriate application (or, misuse) of the epidemiological method. The 33 items are organized in three categories: A) epidemiology-specific methods/techniques used to foment uncertainty and cast doubt about cause-and-effect (18 items); B) arguments used to delay action, maintain the status quo, and create division among scientists (8 items); and C) tactics invoked to misdirect policy priorities through influence (7 items) [4]. Each of these items is listed in Additional file 1.

As an example of the widespread dissemination of invalid information, using the Toolkit, we examined the misinformation provided by the SHAMISEN consortium [5] on the causal relationship of childhood thyroid cancer in Fukushima Prefecture, Japan, after the Fukushima Daiichi Nuclear Power Plant accident. The SHAMISEN review paper [5] has been cited in Japan as part of a larger body of misinformation.

The SHAMISEN international experts’ consortium was established to review the lessons learned from past nuclear accidents, particularly those occurring at the Chernobyl and Fukushima nuclear power plants [5]. However, the review paper by Clero et al. [5], a special publication issued by SHAMISEN, entitled “Lessons learned from Chernobyl and Fukushima on thyroid cancer screening and recommendations in case of a future nuclear accident,” neither provided sufficient references, nor did it convey important information about the results of thyroid cancer screening after the Chernobyl nuclear power plant accident. Citation of the results of screening conducted in Fukushima Prefecture was also incorrect.

Had the abovementioned review paper elaborated on the Chernobyl experience and the status of ultrasound-based screening for thyroid cancer in Fukushima Prefecture using sufficient and correct information, readers of the SHAMISEN review paper would have come to a completely different conclusion. The purpose of our review is to provide information that the SHAMISEN review paper [5] failed to convey, to point out misinformation in the article using the Toolkit by Soskolne et al. [4], and to help readers and policy-makers reach decisions based on factual scientific evidence.

### Lessons learned from Chernobyl

The SHAMISEN [5] review paper stated that, on the basis of thyroid radioactivity measurements taken immediately after the accident at Chernobyl in 1986, systematic thyroid screening using ultrasound echography was initiated 10–12 years after the accident. However, this gives the reader a false impression. Thyroid screening using ultrasound began in 1990 in Gomel, Belarus [6, 7]. The following year, in 1991, the use of ultrasound for thyroid screening was greatly expanded [6, 7].

Subsequently, as mentioned in the SHAMISEN review paper [5], debate about the screening effect and overdiagnosis began in Chernobyl during the 1990s, which debate is now taking place in Fukushima. Welch and Black [8] defined overdiagnosis as diagnosing a disease that would not cause symptoms or death [8]. Those authors cited two reasons for the overdiagnosis of cancer: 1) the cancer does not progress (or actually regresses), or 2) the cancer progresses slowly enough that the patient dies from other causes before cancer symptoms appear [8]. Although some in Japan distinguish the latter as a screening effect, just as in the SHAMISEN review paper [5], we describe “overdiagnosis” as reasons 1) or 2) above in this paper. The same phenomenon of very slow growth occurs in both situations, so it is not possible to distinguish between the two.

The argument that the thyroid cancer cases identified in Chernobyl using ultrasound echography were not the result of the Chernobyl accident, but rather the result of overdiagnosis, had been ongoing for a long time. In response to this debate, between 1998 and 2000, Japanese researchers, Shibata and colleagues [9], investigated children born after the Chernobyl accident in comparison with children born before the accident, using the same procedure as in previous thyroid screenings using ultrasound echo. The results were clear. A smaller number of thyroid cancers were found in children who were fetuses at the time of the Chernobyl accident than the number detected in children who were already born at the time of the accident; however, no thyroid cancers were found in children born after January 1987. This result was likewise confirmed in other studies conducted among unexposed populations around Chernobyl [10, 11]. In the group with relatively low exposure, screening conducted during the 1990s showed that very few thyroid cancers could be detected via ultrasound screening; the procedure at that time was the same as the ultrasound thyroid examinations currently being conducted in Fukushima [12, 13]. Resolution in approximately 12 years after the Chernobyl accident was 7.5 Mhz and that in Fukushima was 10 Mhz in the screening program [12, 13]. If the resolution is increased, a node should receive a secondary examination only if it is 5.1 mm or larger. Also, a secondary examination may be conducted only if a cyst is 20.1 mm or larger. This rule has remained unchanged for 30 years, since thyroid examination using ultrasound echography was introduced in Chernobyl. Therefore, advances in resolution have hardly changed the number or quality of nodes/cysts greater than 5.1 mm/20.1 mm required to undergo secondary examination [14].

We show the results of thyroid screening in the unexposed and low-exposure groups around Chernobyl in Table 1.

**Table 1:** Thyroid screening via ultrasound echography among populations with no, low, and high exposure around Chernobyl

Author(s)	Age at time of accident	Period of investigation	Age at screening	Study area	Exposure or contamination level	Number of examinees	Thyroid cancer cases detected
Belarus Screening Program ^a^	Born after 1987	2002	Less than 15 y	Gomel	Unexposed in severely contaminated areas	25,446	0
Shibata ^b^	Born after 1987	1998–2000	8–13 y	Gomel	Unexposed in severely contaminated areas	9472	0
Ito ^c^	0–10 y	1993–1994	7–18 y	Mogilev	Relatively low	12,285	0 (2) ^d^
Ito ^c^	0–10 y	1993–1994	7–18 y	Bryanks	High	12,147	0 (8) ^d^
Ito ^c^	0–10 y	1993–1994	7–18 y	Zhitomir	High	11,095	1 (9) ^d^
Ito ^c^	0–10 y	1993–1994	7–18 y	Gomel	High	8949	2 (39) ^d^
Ito ^c^	0–10 y	1993–1994	7–18 y	Kiev	High	10,578	1 (6) ^d^

^a^ Krysenko [11]

^b^ Shibata et al. [9]

^c^ Ito et al. (results from June 1993 to May 1994) [12]

^d^ Values in parentheses are results in 1996 (https://nippon.zaidan.info/seikabutsu/1999/00198/contents/009.htm)

Although information on thyroid testing using ultrasound echography was lacking, a similar trend as in Belarus, with more than 35 mSv, was also observed in Ukraine from 1989 to 2008 [15]. The incidence rate in young age groups (up to 19 years of age) in high-exposure regions (more than 35 mSv) decreased substantially during 2005–2008 when examinees were diagnosed were thyroid cancer, probably because most members of these age groups (and all since 2006) were individuals born in 1987 or later and were not directly exposed to radioiodine (^131^I) from the Chernobyl accident in 1986.

However, the above important publications were not cited in English-language articles published by Japanese and Belarusian researchers that suggested a lack of “overdiagnosis” during the first round of screening (Table 1) [9–12, 15–17].

In unexposed children, the evidence showed that childhood thyroid cancer was rarely detected in thyroid screening using ultrasound echo in Chernobyl; this means that, in fact, the issue of overdiagnosis was practically non-existent. As a result, it was finally agreed that the large number of thyroid cancers detected in thyroid examinations using ultrasound in the vicinity of the Chernobyl nuclear power plant were not from overdiagnoses but were, instead, caused by the nuclear power plant accident. The groundbreaking implications of these study results were recognized, not only by environmental epidemiologists, but also by many who were interested in the effects of radiation exposure. However, these important study findings were not cited in the SHAMISEN review paper. Professor Nagataki, a member of the research team (Sasakawa Memorial Healthcare Foundation Project), later stated the following at a symposium [16] held in Fukushima Prefecture in 2016:

“In 2000, it was difficult to confirm the increase in thyroid cancer in the 10th year because the dose relationship was not clear. As a method to confirm the increase in thyroid cancer, even though the dosimetry was unclear, the Sasakawa Memorial Health Foundation project conducted a survey in Gomel Province on the population of children born before and after the nuclear power plant accident. As a result, no incidence of thyroid cancer was observed in the group born after the accident (the group not exposed to radiation). This confirms that the increase in thyroid cancer was due to the exposure associated with the nuclear power plant accident” [16].

Professor Nagataki also explained this for the Japanese media [17].

The failure to include this quotation in the SHAMISEN review paper [5] demonstrates the misuse of epidemiology in relation to items A15 (Suppressing data), A17 (Biased reporting), and C3 (Failing to generalize health risks) of the Toolkit [4].

Without referencing the results of the studies listed in Table 1, only the study by Hayashida et al. [18, 19] was emphasized in the SHAMISEN review paper [5], as if the former article reported the only screening using ultrasound echography conducted in an unexposed group. Hayashida et al. reported a 1.0% prevalence of ultrasound-detected thyroid nodules > 5 mm or cysts > 20 mm [18], and one case of thyroid cancer, out of 4365 examinees (0.023%; 95% confidence interval 0.00058–0.13) [19]. In comparison with the results of first-round screening in Fukushima, where 115 cancer cases were detected out of 300,473 (0.038%; 95% confidence interval 0.032–0.046) [20], the prevalence in Hayashida et al. was 0.6 times smaller. One study including a small number of participants was included in Hayashida et al. [18, 19]; however, overdiagnosis is far from proven considering the evidence from numerous studies among unexposed groups (Table 1) [9–12].

The papers by Hayashida et al. [18, 19] cited in the SHAMISEN review paper [5] exhibit the misuse of epidemiology in relation to items A2 (Ignoring Type II errors) and A3 (Inappropriately interpreting the statistical analysis or results) of the Toolkit [4].

### IARC technical publication No. 46

In 2017, the International Agency for Research on Cancer (IARC) invited researchers to Lyon, France, to discuss the issue of overdiagnosis and provide their recommendations [21], with funding of JPY 35 million (approximately USD 350,000 or EUR 280,000) from the Japanese Ministry of the Environment. In 2018, the IARC Expert Group on Thyroid Health Monitoring after Nuclear Accidents published its recommendations in IARC technical publication No. 46 [21, 22]. These recommendations were consistent with the SHAMISEN recommendations, and were cited in the SHAMISEN review paper, also ignoring the well-known and important published papers [9–12] indicated above and in Table 1. Instead, as described in the following section, the evidence of overdiagnosis presented in the IARC publication [21] involved ultrasound screening of thyroid cancer among middle-aged and older people. Furthermore, IARC publication No. 46 [21] did not convey that only cancers > 5 mm in diameter were detected in the Fukushima screening program to, in fact, avoid overdiagnosis [8].

In its publication No. 46, the Expert Group recommended against population thyroid screening after a nuclear accident and also recommended that consideration be given to offering a long-term thyroid monitoring program for higher-risk individuals after a nuclear accident. Furthermore, without any evidence, the IARC expert group defined “higher-risk individuals” as those exposed in utero or during childhood or adolescence with a thyroid dose of 100–500 mGy or more [21]. The definition seemed to be contrary to evidence from a population-based registry in Ukraine, which indicated that, in 177 cases of childhood thyroid cancer (ages 0–14 years at the time of surgery) among 345 (51.3%) cases from 1986 to 1997, radiation doses less than 100 mGy were received [23]. Also, in annual sex- and 5-year age-specific data of the Ukrainian cancer registry for the population in all regions of the State Committee of Statistics, remarkable increases in thyroid cancer were observed among both male and female individuals, even those with exposures below 35 mGy [15]. These papers [15, 23] were also not cited in the IARC technical publication [21].

IARC [21] excluded all significant evidence from its references regarding overdiagnosis of childhood thyroid cancer. In the report section entitled “Scientific evidence,” IARC emphasized findings among middle-aged and older people and used these in place of evidence among children. Moreover, the same pattern was repeated five times in the IARC publication.

First, IARC emphasized that the main established environmental risk was radiation but that there were many causes, although the evidence was currently limited, and that there was variation between countries. However, IARC did not indicate the extent of the impact of each cause. IARC then highlighted a paper from Korea [24], which reported findings among middle-aged and older people, and presented the paper as if it was evidence of effects in childhood and adolescence.

Then, IARC provided a definition of overdiagnosis. However, this definition has been disproven for thyroid cancer among children and adolescents in studies that were ignored and not referenced by IARC. IARC listed three factors for overdiagnosis to occur: [8] (i) a reservoir of subclinical disease that is detectable by the screening test, (ii) a mechanism by which the tumors can be identified, and (iii) health care activities that lead to the detection. Then, IARC stated that overdiagnosis of pediatric thyroid cancer comprised all three factors using autopsy cases [25] and cases among middle-aged people, older people, or cases among those aged 18 years or older detected using computed tomography scan [26] or palpation/ultrasound echography [27], as well as recipients of health care services [24, 28–32].

In the third instance of this pattern [21], IARC avoided presenting data for children and adolescents and graphs were used instead, with the goal of presenting findings among middle-aged and older adults. IARC noted that “Over the past 20–30 years, the incidence of thyroid cancer in adults has doubled, tripled, or even increased” [21]. After stating that only a few countries have detailed registries of childhood thyroid cancer [33], IARC [21] then showed that thyroid cancer cases had increased among children and adolescents in five of these countries (Denmark, France, Italy, the United Kingdom, and the United States [US]) with the same trends and patterns over time as those in adults. IARC presented age-adjusted incidence rates for the age group 0–19 years in these five countries. IARC also had data for Japan [33] but did not present it. In 2017, IARC was funded by the Japanese government, so the focus should have been primarily on Japan; however, no data were shown for Japanese children and adolescents. We believe that the reason is obvious: if these data had been presented, it would be clear that the trends and patterns of thyroid cancer among Japanese children and adolescents differed over time from those in the other five countries. Additionally, the vertical axis scale showing the degree of incidence in the English version of the IARC publication [21] is not included in the Japanese version [34]. Thus, the Japanese version of IARC Publication No. 46 [34] was designed to make Japanese people believe that there is an increasing trend of thyroid cancer in the five other countries as well as in Japan, to keep the Japanese public uninformed by providing a false impression.

In the fourth instance of this reporting pattern, also using statistical figures, IARC [21] reported the observed versus expected changes in the age-specific incidence of thyroid cancer per 100,000 women during 1988–2007 for eight countries or regions: the US, France, Italy, England and Scotland, the Nordic countries, Korea, Japan, and Australia [21, 28]. However, the data shown for thyroid cancer were for adults aged 20 years and older. Then, in “Overdiagnosis in pediatric thyroid cancer,” where IARC went from discussing thyroid cancer in adults to autopsy data [35], the youngest case was 18 years old. Consequently, for cases aged 18 years and under, the ultrasound data were limited in the post-Fukushima accident. The other three papers [36–38] that claimed no exposure effect among examinees in the screening program of Fukushima Prefecture could not show evidence of overdiagnosis.

Finally, IARC [21] reintroduced the findings from Korea [24], which were repeatedly highlighted. IARC stated that those data [24] served as “an example of what the impact might be of thyroid screening in the general adult population if it were implemented after a nuclear accident.” However, IARC did not mention that thyroid cancer cases in Korea included a larger proportion of small cancers than cases in Fukushima [20, 24]. They also showed that the incidence of thyroid cancer decreased with the introduction of the guidelines in South Korea but did not impart the fact that thyroid cancer guidelines had already been introduced in Japan before that [13, 14].

Ultimately, IARC [21] failed to demonstrate that thyroid cancers in children and adolescents were frequently overdiagnosed in ultrasound examinations. In their abstract [22], however, IARC added that the objective of the IARC Expert Group was not evaluation of the thyroid examination programs that were implemented after past nuclear accidents (the 2011 accident in Fukushima), or recommendations related to thyroid health monitoring activities currently in progress (the screening program in Fukushima); IARC did go on to recommend against population thyroid screening after a nuclear accident.

The context of the puzzling recommendation by IARC cannot be understood without considering the following. The purpose of IARC’s Publication No. 46 [21] may have been to cast doubt on the nuclear accident as the cause of the alarming incidence of thyroid cancer in Fukushima and to show that it is far from possible to reach any conclusions [2]. This may also be true for the SHAMISEN review paper [5].

The first author of the SHAMISEN review paper [5], Dr. Clero, was an advisor to the contributors, and three co-authors were also among the 16 authors of the IARC technical publication No. 46 [21]. Before the first 2017 meeting, all IARC expert group members completed a declaration of interests form required of IARC/World Health Organization (WHO) experts in which they were asked to disclose pertinent research, employment, and financial interests [21]. According to the information provided, IARC identified two authors who had a conflict-of-interest (COI), both of whom had participated as experts. Dr. Dominique Laurier reported that his institution, the Institute for Radiological Protection and Nuclear Safety, receives research funding from Areva (a French multinational group specializing in nuclear power) and EDF (a French multinational electric utility company). However, Dr. Laurier did not declare any competing interests in the SHAMISEN review paper as one of its co-authors. In the IARC meeting in 2017, Dr. Geraldine Thomas, another co-author of the SHAMISEN review paper, reported having received support for travel from Tokyo Electric Power Company, Incorporated (TEPCO), which was responsible for the nuclear accident in Fukushima. However, the review paper [5] did not include this conflicting interest for this expert who collaborated in the SHAMISEN consortium.

Since its founding in the 1960s, many governments have relied on IARC as an authority on the identification and classification of carcinogenic hazards to humans, as well as IARC’s published monographs. However, IARC convened a group of experts in Fukushima who had conflicting interests and who were funded by the Japanese government, which promotes a nuclear power policy, and by the Nuclear Safety Research Association, which is supported by 10 Japanese nuclear power companies (with the exception of TEPCO since 2012), nuclear power plant construction companies, nuclear fuel cycle companies, and by the Central Research Institute of Electric Power Industry [39–43].

In its position statement, INEP focused on conflicting interests (COI) because such conflicts are associated with misinformation regarding epidemiological evidence [3]. The effects of the COI within the IARC expert group would lead to the undermining of scientific integrity, the erosion of public trust in the science of epidemiology, and harm to the public in Japan, especially in Fukushima. The IARC meeting and its resultant publication demonstrate the misuse of epidemiology in relation to items A3 (Inappropriately interpreting the statistical analysis or results), A10 (Diluting/washing out/averaging effects in descriptive population comparisons), A15 (Suppressing data), A16 (Failing to recognize information from qualitative evidence), A17 (Biased reporting), B2 (Failing to disclose a conflicting interest), B6 (Reporting findings only in the general population but not in children), C3 (Failing to generalize health risks despite demonstrated effects in humans), C4 (Neglecting to apply or dismissing the precautionary principle when there is evidence to justify interventions to reduce or eliminate risks), C5 (Failing to be transparent in making explicit those value judgments that underlie decisions about selecting appropriate standards of evidence to draw policy-relevant conclusions), C6 (Infiltrating scientific review panels), and C7 (Misdirecting policy priorities through influence) of the Toolkit [4].

### Lessons learned from Fukushima

Six months after the Fukushima Daiichi Nuclear Power Plant accident in March 2011, thyroid screening using ultrasound echography began, in October 2011. The plan for this screening was developed according to thyroid screening procedures following the Chernobyl nuclear power plant accident, as described above. Patients were examined in the same way as in the Chernobyl thyroid examinations [12]. In other words, if a node > 5 mm or a cyst > 20 mm was detected using ultrasound echo at the screening, the patient would undergo a secondary examination and was monitored using ultrasound and other examinations, such as blood biochemical tests. Then, if necessary, fine needle aspiration cytology (FNAC) was performed. If cancer cells were detected in FNAC, surgery was performed, and a pathological diagnosis of cancer was made [20].

**Fig. 1:**
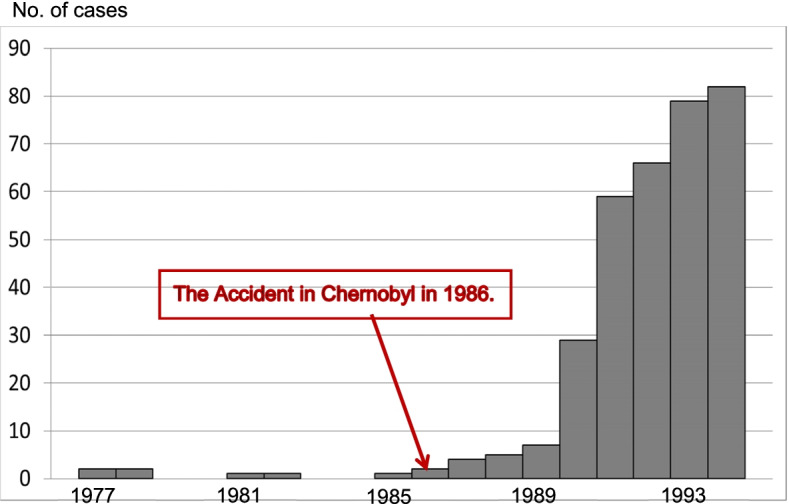
Epidemic curve of childhood cancer in Belarus from 1977 to 1994. Malko MV: Chernobyl radiation-induced thyroid cancers in Belarus. http://www.rri.kyoto-u.ac.jp/NSRG/reports/kr79/kr79pdf/Malko2.pdf.

In the first-round screening using ultrasound that began in October 2011, all residents of Fukushima Prefecture who were aged 18 years and under in March 2011 were included, based on the evaluation of results of thyroid examinations after the Chernobyl accident [20]. In April 2014, after the first round of screening for all eligible individuals was complete, the second round of screening began, with the addition of all children in Fukushima Prefecture who were still in utero when the accident occurred (March 2011) [44]. Children who were not yet fetuses at the time of the accident were not included in the examinations from 2016 [45]. No additional examinees were added in the third round [45]. Screening was conducted in Fukushima in the same way as in Chernobyl, but there were two main differences:

1. The first difference was that in Fukushima Prefecture, screenings began 6 months after the nuclear accident, whereas in Chernobyl, screenings began approximately 5 years after the nuclear accident and were conducted in some areas, but not throughout the region. This was the first experience in the world of thyroid screening using ultrasound up to approximately 5 years after a nuclear accident. Because the Chernobyl experience showed that overdiagnosis rarely occurs (at the time, overdiagnosis was called the “screening effect”) [9–12], it was not foreseen that overdiagnosis of thyroid cancers would occur in Fukushima Prefecture. Therefore, no information about overdiagnosis was given by the prefecture at the planning stage of screening. Thus, at the beginning of the screening program, the prediction made in Fukushima Prefecture was as follows: through the period 2011–2014, only a few cases of thyroid cancer would be detected; subsequently, more cases of thyroid cancer would start to be detected. The period from 2011 to March 2014, was referred to as “preliminary baseline screening,” which was distinguished from “full-scale screening,” conducted after April 2014. However, when the actual examinations were conducted, 115 cases of thyroid cancer were detected by March 2014, which was a large number in excess of the expected number.
2. The second difference was that in Fukushima, to be counted as a case of thyroid cancer, persons must be screened and followed. On the other hand, thyroid cancer cases in Belarus are counted by the Belarus Cancer Registry regardless of whether they have been screened or followed up. The former is an example of more active screening; the latter is an example of more passive screening. This difference in approach to counting cases has led to the claim by the SHAMISEN consortium that in Belarus the minimum latency period of thyroid cancer caused by radiation exposure was 4 to 5 years, while in Fukushima they judged the detection of a large, excess number of thyroid cancer cases in first-round thyroid screening using ultrasound echography from October 2011 to March 2014, as being attributable to overdiagnosis, and not to the accident. However, the number of thyroid cancer cases per year (epidemic curve) after the Chernobyl nuclear accident revealed that the excess incidence of thyroid cancer began in 1987, or 1 year after the nuclear accident (Fig. 1) [46–49]. In one of the studies mentioned above [46], the authors stated that the minimum incubation period was 3 years; however, their table showed that an excess of thyroid cancer began to be observed after 1 year. Therefore, it is possible that the minimum latency period for thyroid cancer in children owing to radiation exposure is 1 year [50], and not 4–5 years.

Thus, thyroid cancers after the Chernobyl nuclear accident could be clinically detected as late as 1990, although thyroid screening using ultrasound echo was not performed in the area around the Chernobyl nuclear power plant at that time. It could be expected that the diameter of thyroid cancers detected using ultrasound echo would be smaller than that of thyroid cancers detected clinically. Therefore, even if the time interval from the accident was 1 year or less, thyroid cancers in Chernobyl would have been detected using ultrasound echo, not only via clinical diagnosis. Therefore, the minimum latency from the accident to identification of nodes > 5 mm by screening should be 1 year or less. Thus, because the first-round screening for thyroid cancer started in Fukushima 6 months after the accident (October 2011 to March 2014), the minimum latency period of 1 year shown in the epidemic curve after the Chernobyl accident [46–49] could be even shorter. These facts indicate that nearly all thyroid cancers detected in thyroid ultrasound examinations in Fukushima were induced by radiation exposure from the accident. The SHAMISEN review paper [5] repeatedly emphasizes “slow” in its discussion of the growth of thyroid cancers, such as “progresses slowly” and “slow growth of most thyroid cancers.” In terms of the latency period, this refers to “mean latency period (or median latency period).” The current issue, however, is the “minimum latency period.”

It should be noted that tumors growing at a rapid rate did not match the definition of overdiagnosis indicated by Welch and Black [8]. Those authors described that even a rapidly growing cancer might still represent overdiagnosis if detected when it is very small; however, this is not the case in Fukushima where cancers less than 5.1 mm were not detected in the screening.

It has been confirmed that nodes containing tumors can stop growing or become smaller. Nodules that stopped growing or became smaller without any clinical symptoms, even if the tumor was growing rapidly, have been observed. All of these have been described and reported by several physicians, and confirmed in video recordings. For example, in the first round, among 1369 cases with nodules larger than 5.1 mm or cysts larger than 20.1 mm, in the second round, the nodules disappeared in 108 cases, became smaller in 530 cases, and remained the same or became larger in 731 cases. If cancer cells were found via FNAC in these 731 cases, the individual underwent surgery.

SHAMISEN [5], IARC [21, 22], and the United Nations Scientific Committee on the Effects of Atomic Radiation (UNSCEAR) [51, 52], have never referred to publications [9–12, 15, 16] showing that nearly no overdiagnosis occurs in thyroid cancer screening of unexposed children. Instead, the data presented as evidence of overdiagnosis [5, 24, 53–57] were all regarding ultrasound screening of thyroid cancer among middle-aged and older people [24, 53–57]. Thyroid cancer in individuals younger than 20 years old has an annual incidence of approximately two cases per 1 million people [58]. However, thyroid cancers in people over age 40 years in Japan have an annual incidence rate of more than two cases per 10,000 [58]. Furthermore, if the 115 cases of thyroid cancer detected during first-round screening [20] in Fukushima were attributable to overdiagnosis, very few cases of thyroid cancer would be expected to be identified in second-round screening because most overdiagnosed cases would have been harvested during the first round. However, 71 cases of thyroid cancer were detected in the second round [44], and the degree of excess in the number of thyroid cancer cases was similar to that in the first round [20]. The ultrasound thyroid examinations conducted by Fukushima Prefecture involve video recordings of all patients who have undergone ultrasound examination, and these recordings have been archived. If thyroid cancer is detected in the second round, the condition in the first round can be confirmed. Of the 71 thyroid cancer cases detected in the second round, 33 showed no nodes or cysts on images in the first round of screening; 25 showed cysts less than 20.1 mm in the first round, 7 had nodes less than 5.1 mm in the first round, 5 had nodes larger than 5 mm and/or cysts larger than 20 mm in the first round, and one case did not undergo first-round examination [44]. Because several physicians judged and confirmed the results, it is unlikely that any cases were overlooked. If such cases had been frequently missed, the number of thyroid cancers detected in the first round would have been increasingly higher. If a thyroid tumor grows at a rapid rate until it reaches 5.1 mm but then stops growing and does not cause any clinical symptoms, then tumors detected in either the first or second round cannot be considered thyroid cancer cases because growth stops while the patient is being followed up in a secondary examination and the patient does not undergo FNAC. Therefore, this is not an example of overdiagnosis. Cancers that grow more than 5 mm in diameter over a 2-year period, such as those childhood thyroid cancers detected in second-round screening in Fukushima, fall outside the category of overdiagnosis [8]. This has also been reported by Kato [59, 60], Yamamoto et al. [61], and Toki et al. [62] However, the SHAMISEN review paper [5] erroneously indicates that no radiation-related risks were demonstrated in those papers [59–62].

Misuses of epidemiology in the SHAMISEN review paper’s [5] introduction on childhood thyroid cancer in Fukushima are demonstrated in relation to items A3 (Inappropriately interpreting the statistical analysis or results), A5 (Failing to allow for adequate follow-up time, partly owing to the inadequate study design by Fukushima Prefecture), A15 (Suppressing data), A16 (Failing to recognize information from qualitative evidence), A17 (Biased reporting), B6 (Reporting findings only in the general population but not in children), and C1 (Assuming that “no data” equates to “no risk”) of the Toolkit [4].

In Fukushima, the first round of screening alone took 2.5 years; since then, residents have been screened every 2 years [20, 44, 45, 63, 64]. In other words, it takes approximately 2 years to complete the entire cycle of thyroid examinations in Fukushima Prefecture. When we consider valid effect estimates, what is important is the time to complete one screening round (2 or 2.5 years), which geographic areas are screened first, and which areas are screened later [20, 63, 64].

The second main difference in screening after the two nuclear accidents is that, in Fukushima, all children and adolescents aged 18 years or less are screened every 2 years, whereas, in Chernobyl [65], there are few plans for all children and adolescents to be screened on a regular basis. In the first round alone, it took 2.5 years to screen all eligible residents of Fukushima Prefecture [20, 44, 45, 63]. This design was decided by the Prefecture, which claimed that radiation less than 100 mSv did not cause cancer and therefore, no excess cancers would be observed. In addition to that, only Fukushima Prefecture directly handled the data of its residents. Researchers had to obtain their information from reports published by Fukushima Prefecture. The Prefecture did not use standard epidemiological analyses, so the information had to be further analyzed to estimate the effects of the accident. Therefore, researchers could not directly adjust for confounding with individual data using methods such as stratification or statistical modeling.

The above actions by Fukushima Prefecture demonstrate misuses of epidemiology in relation to items A5 (Failing to allow for adequate follow-up time), A14 (Inappropriate study design and analytical methods), and A15 (Suppressing data) of the Toolkit [4].

**Fig. 2:**
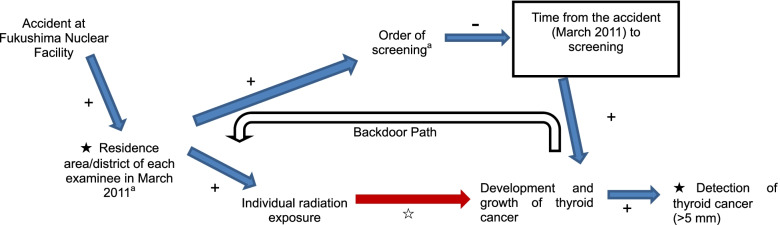
Directed acyclic graph explaining confounding owing to the timing of screening ^a^Screening program was started according to the order of areas of Fukushima Prefecture with the highest contamination levels, determined using WHO Preliminary dose estimation [66] ☆ indicates the primary causal hypothesis in the present study ★ indicates main variables analyzed. Box indicates the adjusted confounding factor. Blue arrows indicate causal paths. The red arrow indicates the main causal path. The white arrow indicates the backdoor path induced by confounding. "+" or "-" with each arrow indicates positive and negative correlation, respectively

The screening order in the first round, which took place from October 2011 to March 2014, began in the area with the highest exposure doses and ended in areas with the lowest doses, using preliminary dose estimation according to the WHO [20, 63, 64, 66]. Thus, screening of areas with the highest doses ended within 1 year after the accident, within which time thyroid cancers would have had little time to develop [20]. According to the screening order, areas with the lowest doses were screened between 2 and 3 years after the accident, with thyroid cancers having more time to develop in these areas than in areas screened earlier. Therefore, confounding was introduced into the screening plan by Fukushima Prefecture; a particular radiation dose affected not only the individual radiation dose but also the order of screening. We have depicted this using a directed acyclic graph (Fig. 2). Areas screened earlier had both a higher radiation dose and shorter time after the accident. Contrarily, the last areas to be screened had both the lowest radiation doses and the longest time after the accident to allow for the development and growth of thyroid cancers. As a result, the lowest likelihood of detecting thyroid cancer was in areas screened earlier, even though these areas had the highest doses and highest potential incidence of thyroid cancer. Conversely, areas screened later had a higher likelihood of detection, even though they received lower radiation doses and had a lower potential incidence of thyroid cancer.

**Fig. 3:**
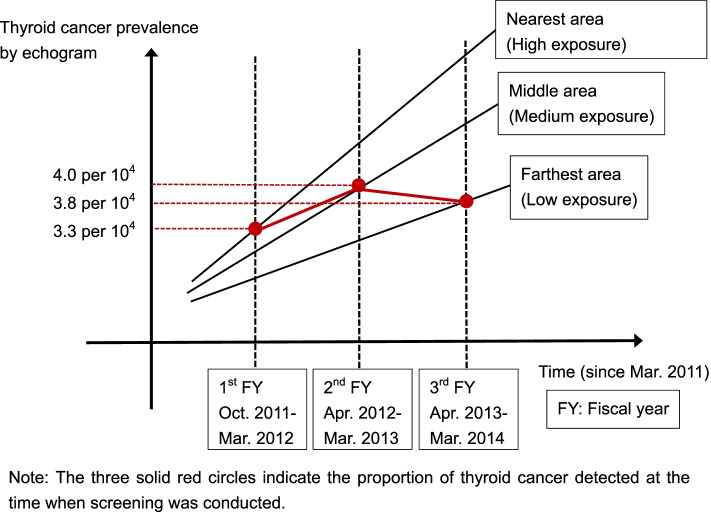
Relationship between elapsed time since the accident and proportion of thyroid cancer detected via ultrasound echography among three screenings in three exposed areas

To complement Fig. 2, Fig. 3 shows the relationship between the growth rate of thyroid cancer and the timing of first-round thyroid screening [20]. Fig. 3 also shows that the detected thyroid cancers were induced by radiation exposure attributable to the accident. Time is represented on the horizontal axis of this figure; it shows the first, second, and third years of the first round of screenings, which took place over a period of 3 fiscal years (FYs), from 0.5 years to 3 years from the time of the accident. The vertical axis in Fig. 3 shows the prevalence of thyroid cancer in the first, second, and third years. If thyroid cancer was induced by radiation exposure attributable to the accident, as shown in the figure, the highest-dose area would have the highest prevalence, the medium-dose area would have the next highest prevalence, and the low-dose area would have the lowest prevalence. The straight line extending in a diagonal direction toward the upper right shows the growth of thyroid cancer in each of the three areas with different exposure levels, which increases with time. The vertical dotted line indicates the time (years) when thyroid examinations using ultrasonic echo to detect these thyroid cancers were conducted. The three intersections of the diagonal straight line and the vertical dotted line (Fig. 3; solid red circles connected by red lines) indicate the prevalence of thyroid cancer at the time of the three screenings in the first round. From this figure, it can be predicted that the prevalence will be low in the first year, high in the second year, and low again in the third year, which adjusts for the confounding effect. The actual observed prevalence was similar to that in Fig. 3, which could be predicted at the beginning of the screening program.

Therefore, confounding induced underestimation toward an inverse effect in the detection of thyroid cancers, which needed to be adjusted by controlling for the time from the accident to screening (Fig. 2). This confounding was the reason for differences in cancer detection between areas with high and low doses that were not apparent in the first screening round (Fig. 3) [20, 63, 64]. The figure depicts the relationship between time elapsed since the accident and the proportion of thyroid cancers detected using ultrasound echography according to the timing in the three screening areas. Hence, in the second round, with longer duration of time since the accident in both regions and smaller ratios to each other, the prevalence among regions better reflected high and low exposure, although there was still some underestimation owing to confounding. This could also explain why thyroid cancers detected with ultrasound echo can be considered to be induced by radiation exposure attributable to the accident. At the initial planning stage of ultrasound thyroid examinations conducted by Fukushima Prefecture, the introduction of such confounding should have been anticipated. At that stage, the Prefecture could have devised ways to prevent the introduction of confounding.

The SHAMISEN review paper [5] ignored the effect of confounding owing to the time interval from the accident to screening on the causal inference between the nuclear power plant accident and childhood thyroid cancer in Fukushima. This corresponds to the misuse of epidemiology in relation to items A3 (Inappropriate interpretation of the statistical analysis or results), A10 (Diluting/washing out/averaging effects in descriptive population comparisons), and A14 (Inappropriate analytical methods in the analysis) of the Toolkit [4].

**Fig. 4:**
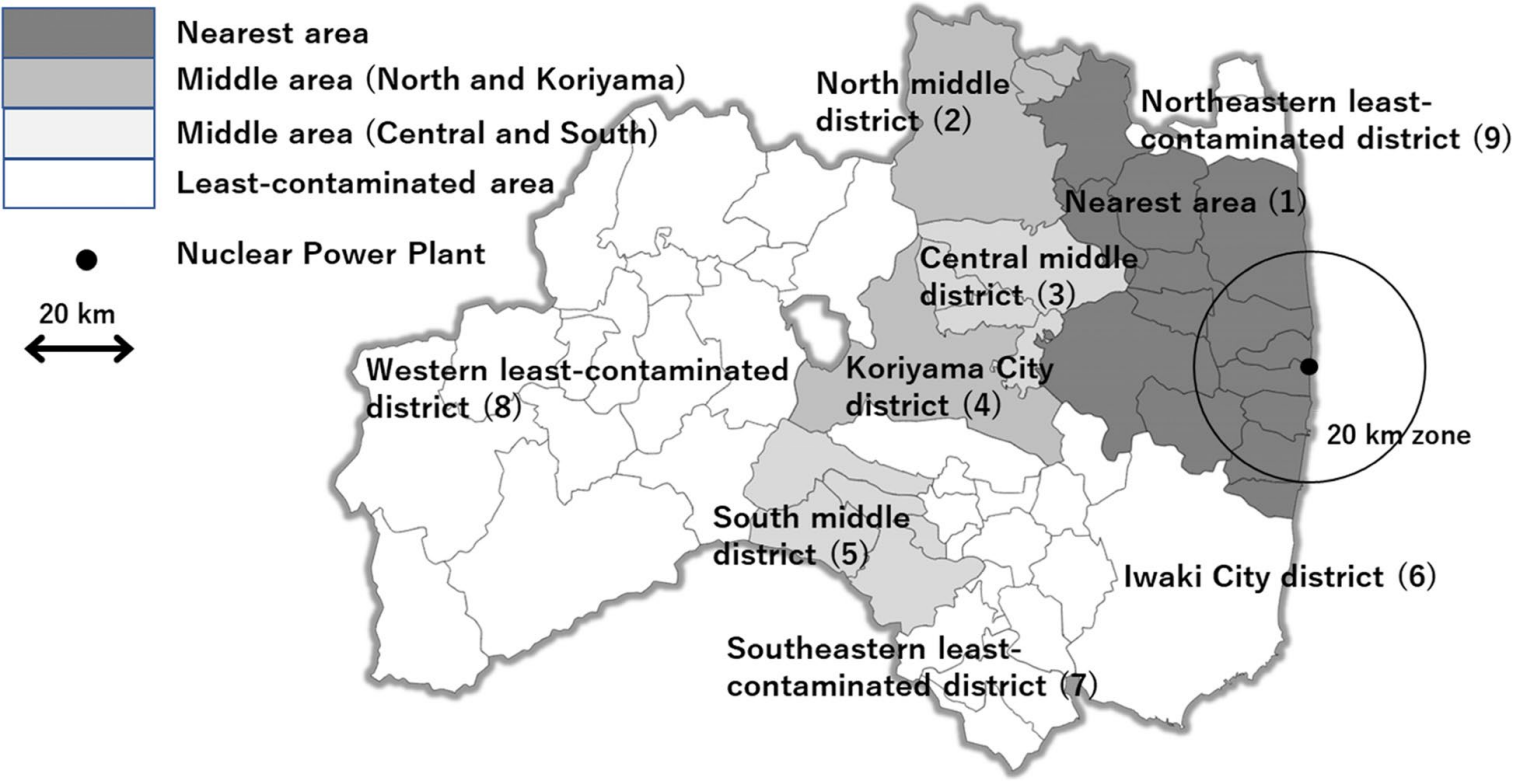
Map of Fukushima Prefecture and its screening areas/districts for analysis

Unlike in the first round [20, 63, 64], the time from the accident to the second round of screening was 2 years [44]. Thus, in the second round, the difference in duration from the accident to screening between the highest-dose and lowest-dose areas became smaller than that in the first round because 24 months were added to both durations in the highest-dose and lowest-dose areas in the previous round. Thus, underestimation induced by confounding owing to the duration until screening in the second round became smaller than that in the first round [20, 44, 63, 64]. As a result, differences in the prevalence odds ratios (PORs) and standardized incidence ratios (SIRs) of thyroid cancer among areas in the second round became more evident than those in the first round. Although these are unadjusted estimates of confounding (Table 2), we show the results of both internal and external comparisons of the first and second rounds using PORs and SIRs, respectively, in each area/district of Fukushima Prefecture in Fig. 4.

**Table 2:** Prevalence odds ratio and age-standardized incidence rate ratio in the first and second screening, Fukushima

Area and district		1st-round screening ^a^					2nd-round screening			
		Internal comparison		External comparison			Internal comparison		External comparison	
	Cases^d^/	POR ^b^	(95% CI)	SIR ^c^	(95% CI)	Cases^d^/	POR ^b^	(95% CI)	SIR ^c^	(95% CI)
	Examinees					Examinees				
(1) Nearest area	14/41,810	1.1	(0.5, 2.7)	37.1	(20.3, 62.3)	17/34,558	3.5	(1.2, 12.0)	60.5	(35.2, 96.8)
(2) North middle district	12/50,617	0.8	(0.3, 1.9)	28.1	(14.5, 49.0)	11/45,580	1.7	(0.6, 6.2)	35.7	(17.8, 63.9)
(3) Central middle district	11/18,193	2	(0.8, 5.0)	75.8	(37.9, 135.7)	4/16,346	1.7	(0.4, 7.6)	38.3	(10.4, 98.0)
(4) Koriyama City district	25/54,062	1.5	(0.7, 3.5)	62.2	(40.2, 91.8)	18/48,046	2.6	(0.9, 9.1)	57.3	(34.0, 90.6)
(5) South Middle district	8/16,465	1.6	(0.6, 4.3)	62.6	(27, 123.3)	2/14,637	1	(0.1, 5.4)	22.1	(2.7, 79.8)
(6) Iwaki City district	24/49,430	1.6	(0.8, 3.6)	67.2	(43.0, 99.9)	9/45,265	1.4	(0.4, 5.2)	25.7	(8.0, 41.2)
(7) Southeastern least- contaminated district	9/29,816	1	Reference	48.3	(22.1, 91.7)	4/28,088	1	Reference	21.7	(5.9, 55.5)
(8) Western least-contaminated district	12/33,720	1.2	(0.5, 2.9)	62.9	(32.5, 109.9)	5/32,208	1.1	(0.3, 4.6)	22.9	(7.4, 53.4)
(9) Northeastern least-contaminated district	0/6360	0	(0, 1.9)	0	(0, 123.0)	1/5788	1.2	(0.05, 9.7)	27.3	(0.69, 152.1)
Total	115/300,473	1.3	(0.7, 2.7)	–	–	71^e^/270,516	1.8	(0.7, 5.9)	–	–

*Abbreviations*: *POR* Prevalence odds ratio, *SIR* Standardized incidence rate, *CI* Confidence interval, *FNAC* Fine needle aspiration cytology

^a^ Data of first-round screening were included in our paper (Tsuda et al., 2016 [63]) up to December 31, 2014. However, in the analysis, the data were up to March 31, 2017 [20, 63]

^b^ Prevalence odds ratio (internal comparison)

^c^ Age-standardized incidence ratio compared with the Japanese national cancer registry from 2001 to 2008 (external comparison)

^d^ FNAC-positive patients (i.e., those in whom cancer cells were detected via cytology) nearly always had histologically confirmed cancer; therefore, we counted FNAC-positive patients as cancer cases in Table 2

^e^ Of the 71 thyroid cancer cases in the second round, 33 had no nodes or cysts on images in the first round of examination; 25 had cysts less than 20.1 mm in the first round; 7 had with nodes less than 5.1 mm in the first round; 5 had node(s) larger than 5 mm and/or cyst(s) larger than 20 mm in the first round; and 1 case did not undergo first-round examination [44]

# All the data necessary to reproduce the results reported in Table 2 are available from the Fukushima Prefecture website. The reference number in the text is 20 for first-round screening as of March 31, 2017 and 44 for second-round screening as of June 30, 2017.

[20] Fukushima Prefecture. Thyroid ultrasound examination (Preliminary baseline screening): Supplemental Report of the FY Survey. Materials and Minutes of Prefectural Oversight Committee Meetings. Reported on 5 June, 2017. http://kenko-kanri.jp/en/health-survey/document/pdf/27_5Jun2017.pdf. Accessed 22 Oct 2020

[44] Fukushima Prefecture. Thyroid ultrasound examinations (First full-scale Thyroid Screening Program). Materials and Minutes of Prefectural Oversight Committee Meetings. Report of Second-Round Reported on 23 October 2017. http://kenko-kanri.jp/en/health-survey/document/pdf/28_23Oct2017.pdf. Accessed 22 Oct 2020

### Exposure measurements in Fukushima

In Chernobyl, measurements of radioactive iodine were taken immediately after the accident [5], but in Fukushima, very little measurement was done [67]. The Nuclear Safety Commission of the Cabinet Office estimated doses using the System for Prediction of Environmental Emergency Dose Information, which indicated that the equivalent thyroid dose from March 12 to March 24, 2011, was over 1000 mSv up to 10 km from the nuclear power plant, and over 500 mSv up to 20 km away [66].

Despite a lack of assessment within 20 km of the nuclear power plant [66], the WHO estimated thyroid equivalent doses in 2011 to be 100–200 mSv for infants in the most-affected areas that were 20–30 km from the nuclear power plant, and 10–100 mSv in the rest of Fukushima Prefecture, as delivered by inhalation, external exposure from ground shine, and ingestion [66]. The estimate of 100–200 mSv for infants in the most-affected areas was 300–1000 mSv in the draft, and 10–100 mSv for infants in Tokyo and Osaka. Later, the Japanese Ministry of Health, Labor and Welfare demanded a revision, and the WHO subsequently lowered the estimates [68].

The National Institute of Radiological Sciences (NIRS) estimated equivalent doses in mothers and infants using the data of Unno et al. [69], with 119 samples collected from late April to early May in 2011 and using an acute ingestion model [70, 71]. These 7 out of 119 estimated doses ranged from 119 to 432 mSv in mothers and from 330 to 1190 mSv in their infants for those living 45 to 220 km south or southwest, including Iwaki City in Fukushima Prefecture, Ibaragi Prefecture, and Chiba Prefecture.

Radiation doses among 1080 children under the age of 15 years were measured by the NIRS in Iwaki City (134 children), Kawamata Town (647 children), and Iitate Village (299 children) in Fukushima Prefecture from March 24 to 30, 2011 (the so-called 1080 survey) [72–75]. No children showed a level greater than the 0.2 mSv/h threshold (equivalent to 100 mSv); the highest level was 0.1 mSv/h (equivalent to 50 mSv) [75]. In addition to a small number of measurements in a limited geographic area with significant uncertainty, the results of the survey of 1080 children were likely underestimated for several reasons: [75, 76] use of a less-sensitive survey meter, measurement with high background levels, and subtracting the radiation level at the individual’s shoulder—rather than the air-dose level—as the background level, leading to over-subtraction [59].

The survey of 1080 children and direct measurement of ^131^I in mothers’ breast milk by Unno et al. [69] were the only direct measurements after the Fukushima disaster; however, these were done only for the evacuated area and neighborhood prefectures. Later (in 2013) [77], UNSCEAR estimated the settlement-average absorbed doses to the thyroid during the first year for 1-year-old infants evacuated from localities in Fukushima Prefecture to be 15–83 mGy. Then, in 2020, UNSCEAR lowered the estimate to 2.2–30 mGy [52].

After the Chernobyl accident, UNSCEAR also derived the official dose estimates from Chernobyl using a special method that had been applied in other similar situations [78]. UNSCEAR used a simplified assumption of a very tiny dose for its theoretical calculation and then drew the conclusion that such a small exposure was unable to generate statistically observable effects and that any detected cancers would have causes other than radiation. This is the same as what is being done now in Fukushima.

The half-lives of various radioiodines are all too short to be suitable for accurate estimation of exposure concentrations, so the idea of analysis of instrumental variables (IVs) and natural experiments is also important in this respect. In testing such hypotheses, methods using IVs, which are often used in the field of environmental epidemiology, or methods based on natural experiments [79–81], rather than the detailed and precise exposure estimates upon which UNSCEAR are exclusively focused, seem to be sufficient, as indicated in Table 2.

The Japanese Ministry of Foreign Affairs (Disarmament, Nonproliferation, and Science Department, International Nuclear Cooperation Office) voluntarily contributed funds to UNSCEAR under a non-ODA (non-official development assistance) framework for the preparation of its post-Fukushima Daiichi Nuclear Power Plant accident report [82]. The purpose of the contribution was “to support the preparation of the report by UNSCEAR, to hold briefing sessions on the report in Japan, and to implement other projects related to radiation effects.” The amount contributed was approximately USD 863,000 for FY2013 [83]. Additionally, the FY2017 supplemental budget provided approximately USD 650,000 for the preparation of a revised version of the Fukushima report [84]. UNSCEAR is introduced on the Japanese Ministry of Foreign Affairs website as follows: “UNSCEAR conducts surveys and evaluations of the effects of radiation on humans and the environment from a scientific and neutral standpoint, and reports a summary of its findings annually to the United Nations General Assembly and publishes a detailed report every few years” [85]. We suggest that this may not be true.

Results of Fukushima Prefecture’s measurement of radioactive materials in the vegetable, spinach, from March 17 to 21, 2011 were released in 2021. Assuming that a 5-year-old child consumed 250 g of spinach every day until March 20, 2011, the radiation exposure to ^131^I would exceed 100 mSv [71, 86]. The released measurement data supported the results of Unno et al. [69] mentioned above [86]. Owing to the large gap among the estimates above, ranging from less than 1 mSv to more than 1000 mSv, alternative measurements are needed, such as the incidence of childhood thyroid cancer, which is very rare in unexposed populations.

The SHAMISEN review paper [5] did not mention this point at all, even though this large range of dose estimates was reported in Fukushima. The article only mentioned a maximum thyroid equivalent dose on the order of 65 mGy among 1-year-old children for internal radiation and a thyroid dose on the order of 3 mGy for external radiation in Fukushima [5]. However, after the Chernobyl nuclear accident in Ukraine, in 88.7% of thyroid cancer cases among children aged 0–14 years between 1986 and 1997, the children were exposed to thyroid doses less than 1 Gy (15.6% were exposed to less than 10 mGy, 36.2% to less than 50 mGy, and 51.3% to less than 0.1 Gy) [23]. The SHAMISEN review paper [5] emphasized the maximum thyroid dose of 10 Gy or more as the Chernobyl thyroid dose [5]. Although information on thyroid testing using ultrasound echography was lacking, a similar trend to Belarus, with 35 mSv and under, was also observed in Ukraine from 1989 to 2008, as mentioned above [15].

In its position statement, INEP focused on conflict-of-interest (COI) because such conflicts are associated with misinformation regarding epidemiological evidence [3]. The effects of COI among members of the UNSCEAR committee would lead to the undermining of scientific integrity, the erosion of public trust in the science of epidemiology, and harm to the public in Japan, especially in Fukushima.

Indeed, UNSCEAR and its resultant publication exhibit the misuse of epidemiology in relation to items A1 (Relying on statistical hypothesis testing; using “statistical significance” at the 0.05 level of probability as a strict decision criterion to determine the interpretation of statistical results and drawing conclusions), A10 (Diluting/washing out/averaging effects in descriptive population comparisons), A13 (Using inadequate or insensitive laboratory methods, measurement practices, or instrumentation), A15 (Suppressing data), A17 (Biased reporting), B2 (Failing to disclose a conflicting interest), B6 (Focusing on studying and reporting only general population effects to the detriment of identifying and protecting from adverse health impacts the most vulnerable, chemically sensitive, and genetically susceptible individuals, including children and pregnant women), B7 (Demanding an unusually high degree of certainty for the public health problems to be addressed; claims that more data are needed for “proof” of elevated risks; rejection of the precautionary principle), C2 (Failing to study a critical public health issue because of political influence, financial interests, or influence of special interest groups resulting in a repression bias), C3 (Failing to generalize health risks despite demonstrated effects in humans), C4 (Neglecting to apply or dismissing the precautionary principle when there is evidence to justify interventions to reduce or eliminate risks), C5 (Failing to be transparent in making explicit those value judgments that underlie decisions about selecting appropriate standards of evidence to draw policy-relevant conclusions), C6 (Infiltrating scientific review panels), and C7 (Misdirecting policy priorities through influence) of the Toolkit [4].

The SHAMISEN [5] exposure estimation owing to the nuclear accident in Fukushima exhibits misuse of epidemiology in relation to items A3 (Inappropriate interpretation of the statistical analysis or results), A15 (Suppressing data), and A17 (Biased reporting) of the Toolkit [4].

Owing to the statement, “There is general agreement that epidemiological methods used for the estimation of cancer risk do not have the power to directly reveal cancer risks in the dose range up to approximately 100 mSv,” in Annex A (A86) of the International Commission on Radiological Protection (ICRP) 2007 Recommendation [87], Japanese people have been led to believe that there is no increase in cancer owing to exposure below 100 mSv [88]. Some Japanese people have also been led to believe that the detection of excess thyroid cancers in Fukushima must be attributable to overdiagnosis, as reported in the SHAMISEN review paper [5] and IARC recommendations [21, 22], because radiation doses in the Prefecture were reported to be well below 100 mSv.

It must be noted that many research papers [88–90] have shown an increased risk of cancer owing to exposure below 100 mSv, as in the study of fetal exposure to diagnostic radiation by Stewart et al. in 1956, which was cited later in a quantitative review by Doll and Wakeford [89]. A recent meta-analysis showed an increased risk of solid cancers owing to radiation below 100 mSv [90]. The ICRP 2005 Publication 99 emphasized this as being approximately 10 mGy [91]. This contradiction means that, with no evidence from the ICRP 2005 Publication 99 [91], 10 mGy was somehow converted to 100 mSv in the ICRP 2007 Publication 103 [87]. This scientifically unsubstantiated change by an international organization with respect to the amount of radiation exposure for carcinogenic effects has had a strong impact on the overdiagnosis hypothesis.

The ICRP 2007 Publication 103, in its Annex A on carcinogenic effects with less than 100 mSv radiation exposure, exhibits the misuse of epidemiology in relation to items A2 (Ignoring Type II errors), A3 (Inappropriate interpretation of the statistical analysis or results), C3 (Failing to generalize health risks despite demonstrated effects in humans elsewhere), C4 (Neglecting to apply or dismissing the precautionary principle when there is evidence to justify interventions to reduce or eliminate exposures), and C7 (Misdirecting policy priorities through influence) of the Toolkit [4].

### Pathological findings in Fukushima

Pathological findings indicated that thyroid cancers detected using ultrasound echography were not pseudo cancers attributable to overdiagnosis but were true thyroid cancers. This evaluation arose from evidence that a large proportion of thyroid cancers detected via ultrasound in Fukushima showed the characteristics of cancer, namely, metastasis and invasion [92].

Among 115 cases of childhood thyroid cancer found in screening, pathological findings showed that 42.1% had extra-thyroidal invasion, 73.0% had lymphatic and vascular invasion, 80.0% had lymph node metastasis, and 2.6% had distant metastasis. These were not markedly different according to whether the patient was diagnosed less than 4 years after the accident or more than 4 years later (Table 3) [92]. However, all of these cases were said to be overdiagnoses. It is very dubious that extrathyroidal invasion, lympho-vascular invasion, lymph node metastasis, and distant metastasis would be found in a high percentage of “overdiagnosed” cases [8].

**Table 3:** Pathological findings among 115 cases of papillary thyroid cancer detected using ultrasound echography [92]

Pathological change	Less than 4 years after the accident		4 years or more after the accident		Total	
	Number of cases	Percentage	Number of cases	Percentage	Number of cases	Percentage
All papillary carcinomas cases	78	67.8% ^a^	37	32.2% ^a^	115	100% ^a^
Intrathyroidal spread	36	46.2% ^b^	20	54.1% ^c^	56	48.7% ^a^
Extrathyroidal extension	34	43.6% ^b^	14	37.8% ^c^	48	42.1% ^a^
Lymphatic/vascular invasion	56	71.8% ^b^	28	75.7% ^c^	84	73.0% ^a^
Lymph node metastasis	61	78.2% ^b^	31	83.8% ^c^	92	80.0% ^a^
Distant metastasis	3	3.8% ^b^	0	0.0% ^c^	3	2.6% ^a^

^a^ Percentage among 115 cases

^b^ Percentage among 78 cases

^c^ Percentage among 37 cases

Overdiagnosis was also discussed by the Fukushima Prefecture review committee. Professor Suzuki, who wrote the report on pathological findings among cases of childhood thyroid cancer detected in screening [92], opposed the overdiagnosis theory from his direct experience of surgeries among these cases. Professor Suzuki emphasized that he carefully followed up patients detected using ultrasound echography, which he ascertained would not lead to overdiagnosis. After he refuted the idea of overdiagnosis, Professor Suzuki was removed from the Fukushima Prefecture review committee.

The SHAMISEN review paper’s [5] omission of pathological findings regarding childhood thyroid cancers in Fukushima is another demonstration of the misuse of epidemiology in relation to item B3 (Ignoring information suggestive of adverse effects) of the Toolkit [4].

Since the 1990s in Japan, in cases where thyroid cancer is suspected on ultrasonography, FNAC has been performed only in cases where the tumor is greater than 3–5 mm in diameter [12, 93]. “Active follow-up” [93] for adults has also been conducted since 1993, in which thyroid cancers < 10 mm in diameter with no metastasis or other low-risk cases are examined periodically without surgery, and surgery is performed only when signs of metastasis are detected. In 2021, the Japan Thyroid Association expressed its position with respect to suppressing the theory of overdiagnosis in Fukushima by proposing the “active follow-up” of thyroid screening [94]. Professor Suzuki argued that the possibility of overdiagnosis was extremely low and that only conventional clinical cancers were treated according to various criteria whereas the possibility of radiation effects was unlikely [95].

### Critique of our 2016 paper by the SHAMISEN consortium, and our response

In the SHAMISEN review paper [5], our study [63] was classified as an ecological study in which “the authors did not acknowledge the issue of ecological fallacy.” However, the design and method of thyroid testing in the Fukushima Prefectural Health Survey was conducted by Fukushima Prefecture, not by us. We wrote our paper [63] based on the figures in a report prepared by Fukushima Prefecture; we analyzed results collected by the Prefecture and made those available to the public. On October 23, 2015, the Center for Radiation Medicine and Public Health of Fukushima Medical University announced that the prefectural screening programs were “scientifically designed as a cohort study on the effects of low-dose radiation exposure” [96]. We also do not consider that study to be an ecological study, but rather a cohort (prospective follow-up) study [96], despite the fact that the tracking was incomplete. The shortcomings of the cohort study designed by Fukushima Prefecture include confounding.

Our paper [63] considered the effects of radiation exposure in Fukushima to be interregional effects; in fact, studies [61, 59 ,60, 97–103], cited in the SHAMISEN review paper also reported these as inter-regional effects. According to the logic followed in the SHAMISEN review paper [5], these studies might all be ecological studies. Regional exposure data for the air dose at representative sites and evacuation sites were made public, but these were only estimates of the regional exposures. We used the distance from the nuclear power plant as an IV of regional exposure, which might lead to a quasi-experiment [80]. Conventionally, the IV has been used for intention-to-treat analysis in clinical trials and is also used in natural experiments [104]. Therefore, we can say that all studies, including ours [63], were analytical, observational studies (cohort studies) of regional exposure and regional effects.

The authors of the SHAMISEN review paper [5] misunderstood the differences in study design between cohort studies and ecological studies and the difference in analytical methods among the cohort studies. This demonstrates the misuse of epidemiology in relation to items B1 (Insisting on the erroneous application of “criteria of study design” in interpreting the weight of evidence) and B7 (Demanding an unusually high degree of certainty) of the Toolkit [4].

Without giving any reasons, another criticism in the SHAMISEN paper [5] was that data from thyroid cancer screening in Fukushima were not directly comparable with data from national cancer registries that do not use ultrasound echography for cancer detection. However, in studies of cancers caused by occupational and environmental exposures, external comparisons using national cancer data, as well as internal comparisons, are popular and commonly used methods [105]. Because overdiagnosis of childhood thyroid cancer rarely occurs in thyroid screening using ultrasound echo, as explained above [9–12], the question arises of why these data would not be considered comparable. Several published studies have put an end to the controversy surrounding overdiagnosis in Chernobyl [16, 17]. Both Jacob [6] and Katanoda [106], cited in the SHAMISEN paper [5], directly compared data from thyroid cancer screening in Fukushima with data from the national cancer registry. This is truly a direct comparison with national statistics. We wonder why this is acceptable for Katanoda and Jacob, but not for us? Katanoda reported a 20- to 30-fold excess of thyroid cancer compared with national cancer registry data [106]. This was close to the excess estimated in our study [63, 64]. However, Katanoda suggested “the possibility of overdiagnosis” owing to “existing knowledge about the effect of radiation on thyroid cancer,” without providing any evidence [106]. As mentioned above, data from the national cancer registry, which do not include results of ultrasound screening for cancer detection, reflect the annual incidence of childhood thyroid cancers, that is, approximately two cases in 1 million; the incidence of thyroid cancer was several orders of magnitude higher in the areas closest to the site of the Fukushima nuclear power plant accident.

The SHAMISEN review paper [5] ignored the possibility of contamination within Fukushima Prefecture and denied external comparisons. This exhibits the misuse of epidemiology in relation to items A7 (Contaminating controls), A8 (Failing to statistically analyze or account for a broad range of exposure characteristics), and A14 (Inappropriate analytical methods) of the Toolkit [4].

### Changing of the reporting content and screening program by Fukushima Prefecture

In Fukushima, more thyroid cancer cases than expected were found in the second, third, and fourth screening rounds [44, 45, 107], mainly in areas close to the nuclear power plant and in locations where fallout had occurred. After the third round of screening, announcements of the number of thyroid cancer cases detected by the municipality were discontinued, and thyroid cancers detected in Fukushima Prefecture were divided across four regions. From the subsequent fifth round, Fukushima Prefecture has stopped releasing the number of detected thyroid cancers in the above four regions.

To cope with the increasing number of thyroid cancers detected in Fukushima, attempts are being made to reduce the number of residents willing to undergo screening. The SHAMISEN paper emphasizes fear and anxiety among the parents of examinees [5]. In its conclusion, the SHAMISEN review paper revealed a fundamental misunderstanding that mass screening in Fukushima was being carried out with no regard for the wishes of children and adolescents who are scheduled to be screened [5]. In the screening program for thyroid cancer, examinees are provided with information that includes an explanation of the possibility of finding diseases that would otherwise have gone unnoticed for the rest of their lives. Furthermore, contrary to the information given by the SHAMISEN consortium, 93.4% of parents and 88.5% of medical personnel in Fukushima Prefecture have explicitly stated that the screening program should be continued in the future [108].

The SHAMISEN review paper [5] ignored the actual state of screening in Fukushima during the post-nuclear accident period, during which time many cancers were growing at a rate of more than 5 mm in diameter over a 2-year period (Table 2). Furthermore, screening was only done for nodules > 5 mm in diameter, which means that nodules smaller than 5.1 mm would not have been detected. In addition, as mentioned above, the SHAMISEN review paper was based on an unsubstantiated overdiagnosis hypothesis and a misguided review of the effect of thyroid cancer screening in adults [21, 24] rather than in children and adolescents.

The SHAMISEN review paper [5] ignored the possibility of contamination within Fukushima Prefecture and denied external comparisons. This exhibits the misuse of epidemiology in relation to items A15 (Suppressing data), A16 (Failing to recognize information from qualitative evidence), A17 (Biased reporting), and B6 (Reporting findings in the general population and not in children) of the Toolkit [4].

As of June 2021, 266 cases of thyroid cancer have been reported by Fukushima Prefecture [107, 109–111]. However, it has been pointed out that, even among individuals who have undergone screening, many cases of thyroid cancer remain uncounted [109–112]. In addition to previously unidentified cases at Fukushima Medical University [112], cases within and outside the prefecture have been identified through the cancer registry of the National Cancer Center [111]. It is emphasized that, in total, more than 300 cases of thyroid cancer in children and adolescents have already been detected [109–112].

### Tallying Toolkit items, and recommendations for Toolkit enhancement

Although we adopt a slightly modified interpretation in the present commentary, the SHAMISEN review paper [5] demonstrates the misuse of epidemiology in relation to 20 of the 33 items comprising the Toolkit [4], as follows: Part A (Through biased study designs and measurements producing invalid science, fomenting uncertainty, and casting doubt about cause-and-effect), 10 out of 18 items; Part B (Arguments used to delay action, maintain the status quo, and create division among scientists), 4 of the 8 items; and Part C (Tactics invoked to misdirect policy priorities through influence), 6 out of 7 items. Among the 20 items detected as being misuses of epidemiology in the SHAMISEN review paper [5], 12 items correspond to IARC and the Japanese government, which organized and funded the IARC meeting, 5 items correspond to the ICRP, and 3 items correspond to Fukushima Prefecture, although there is considerable overlap.

We recommend that the Toolkit [4] could be enhanced for its more direct application if Part A could be rearranged according to the procedures by which epidemiological studies are actually conducted; that is, the process of developing the individual epidemiological study designs; conducting the epidemiological study; analyzing the data; and reporting the results, including the findings of not only the current epidemiological study, but also other epidemiological studies, mechanistic studies, and other studies used to make decisions [113–115]. We also suggest that the following items could be added to Part A for this purpose: “Deliberate creation of biases, for example, selection, information, and confounding bias,” “Intentionally ignoring or excluding from citation references that should be cited,” “Misinterpretation of exposure and/or disease,” and “Intentionally reducing exposure to cause inconsistency with previous findings or to lead to no effect.”

In the future, with increasing examples of the misuse of epidemiological methods, it will be most informative to create a system by which such examples are accumulated by INEP [3] or by an appropriate agency. Such examples could serve as a reference for assessing the utility of the Toolkit [4] in protecting the public’s health and for its further possible enhancement.

### International cooperation for information sharing between Japan and Europe

In January 2016, International Society for Environmental Epidemiology (ISEE) sent a letter to the governments of Japan and Fukushima Prefecture [116]. The ISEE appealed to both governments to develop a series of measures for scientifically recording and tracking the health status of people in Fukushima Prefecture, which would be useful to better understand and estimate the risks from the 2011 nuclear accident. ISEE also emphasized the need for detailed monitoring of residents’ exposure levels. Additionally, ISEE informed the two governments that it could draw on the expertise of its members as an independent international expert body to support these activities, as needed. However, neither the Japanese government nor Fukushima Prefecture responded to the letter. Moreover, these governments have not investigated any cancers other than thyroid cancer, including leukemia and breast cancer, or non-cancer diseases, the latter having been predicted by the WHO to occur more frequently after a nuclear accident [117].

In the preface of the report “Late lessons from early warnings: science, precaution, innovation,” published by the European Environment Agency (EEA) in 2013 [118], Professor McGlade, Executive Director of the EEA said, “The scientific elites have also been slowly losing public support. This is in part because of the growing number of instances of misplaced certainty about the absence of harm, which has delayed preventive actions to reduce risks to human health, despite evidence to the contrary.” It is suggested that the SHAMISEN consortium ruminate on the meaning of these words.

## Conclusions

We have applied the Toolkit developed by Soskolne et al. [4] for detecting the misuse of epidemiology to the literature of concern with the aim of assessing the impact of misused methods and techniques on scientific discourse and the public’s health. The target was a review paper published in 2021 by the SHAMISEN consortium in *Environment International* that examined and made recommendations regarding past nuclear accidents, particularly those that occurred at Chernobyl and Fukushima [5].

The SHAMISEN review paper emphasized the possible negative health effects of thyroid cancer screening performed in Fukushima and the less aggressive nature of thyroid cancer and its slow progression [5]. The review paper claimed that the cause of thyroid cancer in childhood and adolescence, which has been detected dozens of times more frequently than usual in Fukushima after the severe accident at the nuclear power plant in 2011, was not the result of the accident, but rather the result of overdiagnosis in medical examinations [5]. Furthermore, the review paper did not present important results obtained from thyroid screening using ultrasound echography after the Chernobyl nuclear accident, or evidence negating the possibility of overdiagnosis in Fukushima [5].

Despite *Environment International* encouraging correspondence, we had identified so many issues with the SHAMISEN review paper that we could not have addressed them all in “correspondence”. Therefore, we considered submitting a full article of critique to *Environment International*, but, given that its reviewers accepted the SHAMISEN paper, we reckoned that our article would not likely see the light of day in that Journal. So, instead, we decided to try another journal in the field of environmental epidemiology. However, that Journal rejected our submission recommending that a letter-to-the-editor of *Environment International* would be the more appropriate route to take. This rejection was coincident with the appearance of the Toolkit article in* Environmental Health*. So, we chose rather to submit to this Journal. In so doing, we were able to identify relevant features of the SHAMISEN review paper warranting critique according to the framework provided in the Toolkit. With the Toolkit article, we now had a framework to not only critique the claims made in the SHAMISEN review paper, but also to identify conflicting interests among some of the experts and organizations involved in its authorship.

Our results showed that the SHAMISEN review paper reflects 20 of the 33 items indicating epidemiological misuse from the Toolkit. We believe that our application of the Toolkit is first in assessing the misuse of epidemiology and its impact on health research and health policy. We recommend some additional items for the Toolkit, as well as some reorganization to enhance its utility.

## Supplementary Information

**Additional file 1.** Toolkit of inappropriate applications of the epidemiological method.

## Data Availability

All the data necessary to reproduce the results reported in the submitted paper are available on the Fukushima Prefecture website. References in this paper (20 and 44) are given in the text.

## Abbreviations

COI: Conflicts-of-interest
EEA: European Environment Agency
FNAC: Fine Needle Aspiration Cytology
^131^I: Radioiodine 131
IARC: International Agency for Research on Cancer
ICRP: International Commission on Radiological Protection
INEP: International Network for Epidemiology in Policy
ISEE: International Society for Environmental Epidemiology
IV: Instrumental variable
NIRS: National Institute of Radiological Sciences
POR: Prevalence odds ratio
SHAMISEN: Nuclear Emergency Situations - Improvement of Medical And Health Surveillance
SIR: Standardized incidence ratio
TEPCO: Tokyo Electric Power Company
UNSCEAR: United Nations Scientific Committee on the Effects of Atomic Radiation
WHO: World Health Organization
