# Supplementary material for: Demonstrating the undermining of science and health policy after the Fukushima nuclear accident by applying the Toolkit for detecting misused epidemiological methods
Source: Environ Health. 2022 Aug 24;21:77. doi: 10.1186/s12940-022-00884-6 (PMC9400325; doi:10.1186/s12940-022-00884-6)
Supplement: Supplementary file 1 — Additional file 1. Toolkit of inappropriate applications of the epidemiological method. [file 12940_2022_884_MOESM1_ESM.docx]

**Additional file 1.** Toolkit of inappropriate applications of the epidemiological method. (From Ref. 4)

--- Part A ---

Epidemiology-specific methods/techniques used to foment uncertainty and cast doubt about cause-and-effect [through biased study designs and measurements producing invalid science]

A1 Relying on statistical hypothesis testing; using “statistical significance” at the 0.05 level of probability as a strict decision criterion to determine the interpretation of statistical results and drawing conclusions

A2 Conducting statistically under-powered studies; ignoring Type II errors

A3 Interpreting the statistical analysis or results inappropriately (see B8 below)

A4 Failing to use adequate follow-up methods

A5 Failing to allow for adequate follow-up time

A6 Introducing inappropriate representation of total person-years of exposure, seen

especially in occupational health studies

A7 Contaminating controls

A8 Failing to statistically analyze or account for a broad range of exposure characteristics among the exposed group (cohort studies)

A9 Selecting inappropriate controls; failing to adhere to the requirement that controls

should be representative of the population from which the exposed group (cohort

studies) or the cases (case-control studies) emerged

A10 Diluting/washing out/averaging effects in descriptive population comparisons

A11 Ignoring known synergies among components of a mixture of chemicals

A12 Failing to account for the effects of exposure to complex mixtures in risk assessments

A13 Using inadequate or insensitive laboratory methods, measurement practices, or

instrumentation

A14 Inappropriate analytical methods used in the statistical analysis

A15 Suppressing data

A16 Failing to recognize the validity of evidence from qualitative methods

A17 Producing erroneous or biased meta-analyses and reporting them as representing a weight-of-evidence summary result

A18 Using mortality instead of morbidity data for a cancer endpoint with a high survival

rate

--- Part B ---

Arguments used to delay action, maintain the status quo, and create division among scientists [imposing inappropriate standards and methods of suppression]

B1 Insisting on the erroneous application of “criteria” for causation proposals (e.g.,

Bradford Hill viewpoints or aspects) in interpreting the weight of evidence in a causation analysis to infer causation

B2 Failing to disclose a conflict of interest in the presence of a financial conflicting interest, financial control of agenda-driven funders, political influences, or vested interest goals (see C6 below)

B3 Ignoring mechanistic information suggestive of adverse effects

B4 Exaggerating differences, or dismissing them, when toxicological studies suggest a potential human health hazard

B5 Ignoring related or families of molecular structures that predict potential health

hazards

B6 Focusing on studying and reporting only general population effects, to the detriment of identifying and protecting from adverse health impacts the most vulnerable, chemically sensitive, and genetically susceptible individuals, including children and pregnant women

B7 Demanding an unusually high degree of certainty for the public health problems to

be addressed; claims that more data are needed for “proof” of elevated risks; rejection

of the precautionary principle

B8 Requiring that any observed odds ratio/relative risk between exposure and

disease must have a value of 2 or greater before the study can be admitted to support expert testimony [see A3 above]

--- Part C ---

Tactics invoked to misdirect policy priorities through influence [imposing undisclosed values from the positions taken by special interests]

C1 Assuming that “no data” equates to “no risk”

C2 Failing to study a critical public health issue because of political influence, financial

interests, or the influence of special interest groups, resulting in a repression bias. It must be remembered that the reason some studies are never carried out is not because their approval was not granted; sometimes the reason is because the topic is repressed

C3 Failing to generalize health risks, and restricting the assignment of risk to local populations of exposed people despite demonstrated effects in humans elsewhere

C4 Neglecting to apply or dismissing the precautionary principle when there is evidence to justify interventions to reduce or eliminate exposures

C5 Failing to be transparent in making explicit those value judgments that underlie decisions about selecting appropriate standards of evidence to draw policy-relevant conclusions (i.e., in suppressing dominant interests and values)

C6 Infiltrating editorial boards, scientific review panels, and decision-making bodies of all kinds (see B2 above)

C7 Misdirecting policy priorities through influence
